# The Combination of Immunomodulatory Secretome and Liposome-Bound TRAIL Improves Knee Osteoarthritis Symptoms in an Ovine Model

**DOI:** 10.3390/pharmaceutics18020193

**Published:** 2026-02-02

**Authors:** Joaquín Marco-Brualla, Felícito García-Álvarez, Sara Fuente, Pablo Fernández, Arantza Vitoria, Francisco José Vázquez, Juan Pedro Lapuente-Fernández, Luis Martínez-Lostao, Antonio Romero, Alberto Anel

**Affiliations:** 1 Biochemistry and Molecular and Cell Biology Department, Aragón Health Research Institute (IIS-Aragón), University of Zaragoza, 50009 Zaragoza, Spain; joaquin_marco_91@hotmail.com; 2 Facultad de Farmacia, Universidad San Pablo-CEU, 28660 Boadilla del Monte, Spain; 3 Faculty of Medicine, University of Zaragoza, 50009 Zaragoza, Spain; engaral@unizar.es (F.G.-Á.); lmartinezlos@salud.aragon.es (L.M.-L.); 4 The Agri-Food Institute of Aragón (IA2), Veterinary Teaching Hospital, University of Zaragoza, 50013 Zaragoza, Spain; sfuente@unizar.es (S.F.); avm@unizar.es (A.V.); pvazquez@unizar.es (F.J.V.); 5 Ready for Trading (R4T), Living Cells Molecular and Cell Biology Research Laboratories, Fuenlabrada University Hospital, 28942 Madrid, Spainjplapuente@yahoo.es (J.P.L.-F.)

**Keywords:** osteoarthritis, LUV-TRAIL, secretome, tibial plateau damage, synovial hyperplasia

## Abstract

**Background/Objectives**: Knee osteoarthritis stands as the highest prevalent joint disease worldwide, affecting millions of adults and significantly impairing mobility and quality of life. Pro-inflammatory cells and cytokines are considered key players in the pathophysiology of the disease. In previous work, two anti-inflammatory therapeutic approaches were developed: a secretome enriched in anti-inflammatory cytokines, and nanoliposome-bound TRAIL (LUV-TRAIL), with proven efficacy against rheumatoid arthritis in rabbits. **Methods**: In this work, we evaluated the ability of these treatments to prevent the development of osteoarthritis (OA) in an ovine model following meniscectomies. Two weeks after the surgeries, knees were treated with several rounds of single or combined therapy, and then sheep were left untreated for several months. Knee damage was followed by X-ray analysis and, after sacrifice, assessed through macroscopic inspection, histological determinations, and inflammatory cytokine measurements. **Results**: The combined therapy had a significant positive effect against osteoarthritis development. Specifically, the combination is capable of improving knee injury in the first stages of OA in several parameters, such as synovial hyperplasia and tibial plateau damage, which are two of the most frequently damaged areas. Other markers, such as synovial inflammation and X-ray and macroscopic images, also presented a tendency to improved scores. **Conclusions**: The combination of the secretome with LUV-TRAIL represents a promising therapy worth exploring further in osteoarthritis treatment and/or prevention.

## 1. Introduction

Osteoarthritis (OA) is a chronic, degenerative disease where a joint and its surrounding tissues are progressively damaged, leading to pain and disability in affected patients [1]. Notwithstanding that it may affect any joint in the organism, hip and knee OA are the ones with the greatest social burden. In fact, these disorders represent one of the leading causes of disability worldwide, and their prevalence has steadily increased over the last decades [2]. Although the etiopathogenesis of the disease is complex and multifactorial, there are several well-established risk factors related to OA. In particular, prior trauma in the joint and age have been extensively reported to be associated with the manifestation of this disease [3]. Other features linked with OA are obesity, gender, ethnicity, and even socioeconomic status [4,5].

From a pathophysiological standpoint, this disorder begins with degradation of articular cartilage, which can be detected radiographically as a narrowing of joint space [6]. This damage, along with continued erosion of the structure, leads to more degradation of the subchondral bone and synovium. Other compensatory mechanisms, such as production of cartilage-like tissue, are activated; however, this newly formed tissue will not be as biomechanically as flexible as the previous one, inducing, in turn, pathological synovial hyperplasia [1]. In the knee joint, synovial fluid and meniscus normally function to lubricate the articular space and to absorb the impact of the tibia and femur, respectively. Cartilage degradation will eventually provoke damage in these tissues, featuring loss of synovial fluid and erosion of the tibial plateau and the femoral condyle, among other signs [7].

It is currently known and widely accepted that inflammation plays a central role in the pathogenesis of this disorder [8,9]. Under OA conditions, chondrocytes and synoviocytes can release damage-associated molecular patterns (DAMPs) in the cartilage [10]. These DAMPs can be recognized by synovial fibroblasts and macrophages, which will express in response a multitude of inflammatory cytokines, chemokines, and receptors [11]. This inflammatory milieu promotes the release of metalloproteinases by the aforementioned chondrocytes and synoviocytes, which will degrade the extracellular matrix, leading to more DAMP production and establishing a vicious inflammatory cycle [12]. In addition, many other leukocytes, such as T cells and neutrophils, can be attracted to the joint following this degradation, considered in fact a main feature of OA progression [13,14]. All of this response favors a state of chronic and low-grade inflammation in the joint, slowly and progressively worsening OA’s clinical symptoms.

Unfortunately, there does not currently exist a pharmacological treatment capable of halting or preventing the disease. Beyond lifestyle modifications (diet, exercise, physiotherapy, etc.) and joint replacement, most current options are oriented to alleviating the symptoms of pain by using nonsteroidal anti-inflammatory drugs (NSAIDs) or corticosteroids [15]. Most research efforts are focused on finding alternatives for these therapies, such as intra-articular injections of hyaluronic acid [16], mesenchymal stem cells (MSCs), [17] and platelet-rich plasma (PRP) [18]. Although these approaches have shown promising results in alleviating pain, their efficacy remains still under investigation, and further characterization is needed.

For these reasons, other alternatives to fight OA are being actively explored at present. In this regard, our group has developed a treatment consisting of one-layered nanoliposomes coated with multiple TRAIL molecules on their surface, termed ‘LUV-TRAIL’ (‘LUV’ from Large Unilamellar Vesicles). TRAIL is a death ligand, a member of the TNF superfamily of proteins, that induces cell death on infected or tumor cells while sparing normal cells [19]. It is expressed as a transmembrane protein [20], and it has been demonstrated to be secreted by T cells on the surface of exosomes, a mechanism that enhances its bioactivity when compared to soluble TRAIL [21,22]. Our LUV-TRAIL formulation, which mimics this feature, has proven to greatly enhance cytotoxicity against multiple tumor cell lines in comparison with its soluble formulation [23,24,25]. Concerning normal cells, TRAIL does not induce cell death and, instead, it can trigger NF-κB-dependent survival pathways [26,27], promoting proliferation and viability in vascular endothelial cells [28] or mesenchymal stem cells [29]. The physiological role of TRAIL is rather related to immune regulation [21,30], affecting activated CD8^+^ T cells’ proliferation through the induction of p21 expression [31,32]. Notably, rheumatoid arthritis development has been associated with a decrease in exosomes carrying FasL or TRAIL on their surface within the synovium [33]. In this regard, LUV-TRAIL has been demonstrated to be an effective treatment in a rabbit model for rheumatoid arthritis, substantially reducing inflammation and synovial hyperplasia in rabbit knee joints [34,35] and demonstrating that its immune-regulating function can be exploited in therapy.

Another anti-inflammatory approach has been recently developed, consisting of a secretome from the indirect co-culture of human polarized M2-like macrophages with adipose-tissue-derived mesenchymal stem cells. This incubation allows for interplay and communication between these two cell types, including the secretion of multiple cytokines and factors with a strong anti-inflammatory profile [36]. This secretome has been shown to be effective in both in vitro and in vivo models of severe inflammation induced by LPS [36,37], thus opening the gate for exploring its efficacy in other inflammatory disorders.

In the present study, we sought to evaluate the ability of LUV-TRAIL, the described secretome, or their combined treatment to either delay the onset of knee OA or improve its signs and symptoms. Our results show that the combination of both treatments significantly reduces the development of OA in our ovine model, improving synovial hyperplasia and tibial plateau damage. Other markers, such as inflammation and macroscopic and radiographic scores, also presented a positive tendency that we intend to better characterize in the future.

## 2. Materials and Methods

### 2.1. Generation of Large Unilamellar Vesicles Decorated with TRAIL (LUV-TRAIL)

Formation of lipid nanoparticles with soluble recombinant TRAIL (sTRAIL) anchored to their surface was generated as described in previous studies [23,34]. In brief, the following lipids were mixed in chloroform–methanol (3:1) and prepared at a weight ratio of 50:30:10:5:5: phosphatidyl–choline, sphingomyelin, cholesterol, 1,2-distearoyl-sn-glycero-3-phosphoethanolamine)-N-(methoxy(polyethylene glycol)-2000) (ammonium salt), and 1,2-dioleoyl-sn-glycero-3-[N-(5-amino-1-carboxypentyl)-iminodiacetic acid] succinyl (nickel salt) (Avanti Polar Lipids, Alabaster, AL, USA). To remove the diluent, samples were first dried under a stream of nitrogen gas and subsequently under a vacuum at 37 °C. Next, dried lipids were resuspended in phosphate buffer saline (PBS). In order to obtain unilayered liposomes with a homogeneous size, this suspension was subjected to quick freeze–thaw cycles (at least twice) and forced to flow through two 200 nm pore-sized polycarbonate membranes (Whatman, Maidstone, UK) under pressure using an extruder (Northern Lipids, Burnaby, BC, Canada). This lipid formulation was referred to as LUV.

Recombinant sTRAIL-His6 (amino acids 95-281 with a 6-histidine tag at the N-terminal extreme cloned into the pET-28c plasmic, kindly provided by Dr. Marion MacFarlane) [38] was fixed at the surface of LUVs through incubation of both at 37 °C for 30 min, with gentle shaking, resulting in the formation of LUV-TRAIL.

A fraction of the generated LUVs was kept unmixed for the in vivo experiment at a lipid concentration of 5 mM. LUV-TRAIL preparations were prepared at the same lipid concentration and a final TRAIL amount of 12 μg/mL. Both formulations were filtered by flowing them through 0.22 μm pore-sized PTFE membrane filters (Sigma, St. Louis, MO, USA) using a syringe in a laminar flow cabinet (Telstar, Barcelona, Spain). LUV and LUV-TRAIL were freshly prepared and stored at 4 °C before being injected in animals, with a maximum storage time of one week.

### 2.2. Production and Characterization of the Anti-Inflammatory Secretome 

The secretoeme batch used for these experiments was identical to the one created in previous work [36]. Notably, an erratum in the original article incorrectly interchanged the concentrations of TIMP-1 and GM-CSF; these values are corrected in Table 1.

Manufacturing and protein characterization of this secretome were performed as in previous articles [36,37]. Monocytes were obtained from a bag of donor blood from the Fuenlabrada University Hospital (Madrid, Spain) after peripheral blood mononuclear (PBMC) isolation and cell monolayer formation after a two-hour incubation at 37 °C and 5% CO_2_. They were polarized to M2-like macrophages through incubation with monocyte culture medium (CTS-AIM-V™, Gibco, Waltham, MA, USA; ‘monocyte medium’ from now on) supplemented with 10 ng/mL of M-CSF (R&D Systems, Minneapolis, MN, USA) for 4 days. Adipose-tissue-derived MSCs were obtained from an abdominoplasty from a healthy patient, with previous informed consent, and then isolated, cultured, and characterized at Histocell facilities (Bilbao, Spain), also as described in [36,37]. Cells were cultured until passage 4 and then seeded in six-well plates.

Co-culture was established by seeding M2-like macrophages onto Transwell^®^ inserts (Falcon, PET, 1 µM pore size) (Corning, Corning, NY, USA) and placing them on the six-well plates already containing the adipose-tissue-derived MSCs. They were incubated at 37 °C, 5% CO_2_ in monocyte medium for 23 days; supernatants were collected every 3–4 days, and wells were refilled with monocyte medium. Finally, all supernatants were pooled together, centrifuged at 1800 g for 10 min at 4 °C, filtered, aliquoted, and stored at −80 °C until usage. Aliquots of monocyte medium were also prepared and saved for the in vivo experiment.

As explained above, characterization was already assessed in previous work [36]. Briefly, up to 48 proteins were characterized, 45 of them through Multiplex assay ((ProcartaPlex 45 PLEX, Invitrogen, Grand Island, NY, USA): MIP1-α, SD-1α, IL-27, LIF, IL-2, IL-4, IL-5, IP-10, IL-6, IL-7, IL-8, IL-10, PIGF-1, Eotaxin, IL-12p70, IL-13, IL-17A, IL-31, IL-1RA, SCF, RANTES, IFN-γ, GM-CSF, TNF-α, HGF, MIP-1β, IFN-α, MCP-1, IL-9, VEGF-D, TNF-β, NGF-β, BDNF, GRO-α, IL-1α, IL-23, IL-15, IL-18, IL-21, FGF-2, IL-22, PDGF-BB, VEGF-A, TIMP-1, and MMP-3) and the other three using the ELISA technique (IGF-1, IL-1β, and MMP-1 (DuoSet ELISA kit, R&D, Minneapolis, MN, USA)). A Luminex Labscan 100 (Luminex Corporation, Austin, TX, USA) was employed for Multiplex determination, while an iMark plate reader (Biorad, Hercules, CA, USA) was used for ELISA readings and quantification. Quantitative results are summarized in Table 1.

### 2.3. Animals

Sheep were selected as the in vivo experimental model for knee OA due to the close anatomical and biomechanical similarities to human knees, especially compared to other options [39]. For the in vivo experiment, 32 female sheep were used (breed: rasa aragonesa) without any prior sign or history of injury or disability in their legs. The age and weight of the animals were 16–20 months and 45 kg, respectively. Prior clinical examinations were within standard limits. This study was conducted at the Veterinary Teaching Hospital, belonging to the University of Zaragoza, and in collaboration with the Animal Research Service (SEA, ES502970012006). All procedures were performed under Project License PI01/21, approved by the Ethic Committee for Animal Experiments from the University of Zaragoza. The care and use of animals were performed according to the Spanish Policy for Animal Protection RD53/2013, which meets the European Union Directive 2010/63 on the protection of animals used for experimental and other scientific purposes. All animals, following an accommodation period of two weeks, were kept with food and water ad libitum in the boxes of the aforementioned Veterinary Teaching Hospital and paddocks of the Animal Research Service (University of Zaragoza).

### 2.4. In Vivo Study Design and Procedure

Osteoarthritis symptoms typically emerge several months after surgical destabilization of the selected joint [40]. After evaluation of available surgical approaches, unilateral total meniscectomy was selected to conduct in sheep, as it provides faster, more progressive and consistent joint degeneration [40]. In our study, we sought to assess whether our treatment could not only improve OA signs but also delay the onset of the disease. Therefore, several injections were given to animals before the first signs of OA (see below).

Sheep identification was coded, and they were randomly divided into five experimental groups, with each receiving a different intra-articular treatment:

- LT: 8 sheep, 2 mL containing 12 μg of LUV-TRAIL.
- Secretome: 8 sheep, 2 mL of half-diluted secretome.
- Secretome + LT: 8 sheep, 1 mL of 12 μg/mL LUV-TRAIL + 1 mL of undiluted secertome
- LUV: 4 sheep, 2 mL containing 2.5 mM of LUV.
- Culture media group: 4 sheep, 2 mL dose of monocyte medium.

Prior to surgery, synovial fluid was extracted from a randomly selected forelimb of each sheep for future assessments (termed the ‘pre-op’ experimental point). Partial meniscectomies of the medial meniscus and desmotomies of the anterior cruciate ligament were then performed through arthrotomy under general anesthesia on both forelegs of each sheep. Sheep were allowed a two-week surgery recovery with the adequate antibiotic and analgesic treatment until they were able to walk without any sign of pain or disability. Afterwards, five consecutive treatments were administered through intra-articular injection under sedation once every two weeks. For this purpose, one of the two operated knees was randomly selected for treatment, while the other remained untreated for positive control purposes.

In addition, before treatment injections, a sample of synovial fluid was extracted from every operated knee. They were tagged as pre-T1, pre-T2, pre-T3, pre-T4, and pre-T5 (‘pre-T’ stands for pre-treatment).

After finishing all rounds of therapy, sheep remained untreated for six months. Then, half of the sheep in every group were sacrificed. This first round was named ‘Batch 1.’ From these animals, all treated knees, along with three untreated knees (chosen at random), were further processed for several evaluations (see next sections below). Synovial fluid from the knees was also extracted (named the ‘end-point’ experimental point) immediately before sacrifices.

**Figure 1:**
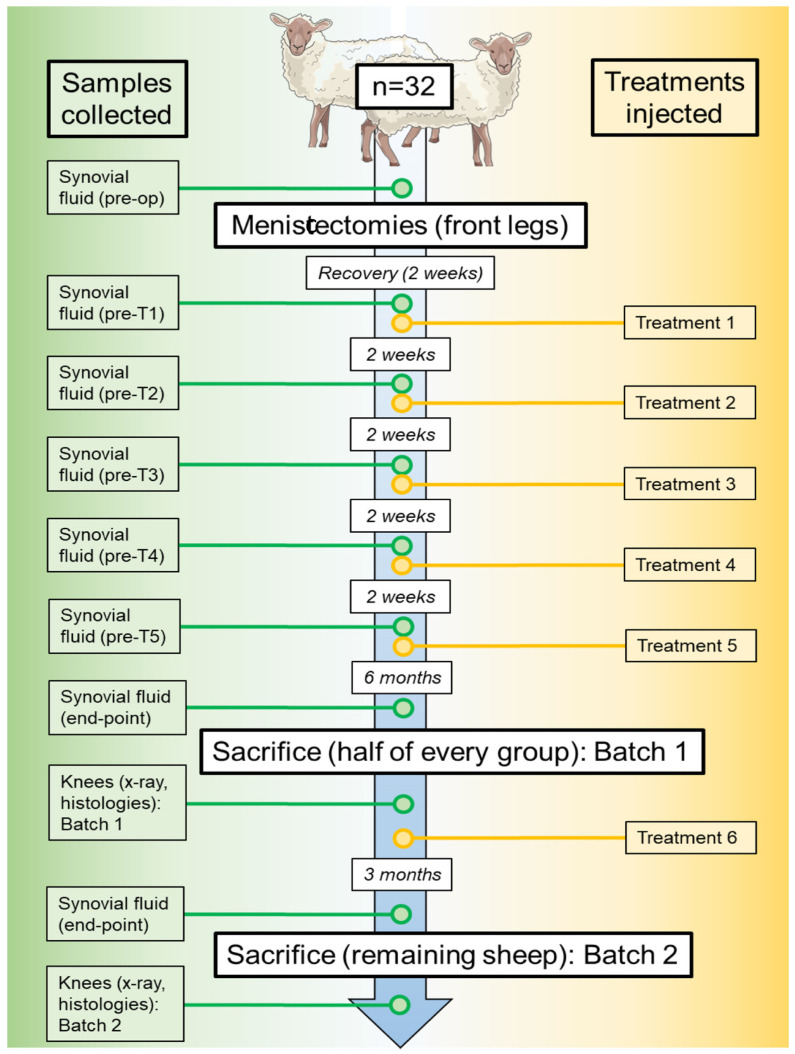
Schematic chronogram of in vivo experiment, including samples collected and treatment timings. Sheep drawing provided by bioicons.com.

The other half of the animals was given one last sixth round of treatments and, three months later, synovial fluid was equally extracted. They were then sacrificed. Similarly to Batch 1, all treated knees and five of the untreated knees were also collected, and synovial fluid was collected for the next assessments. This second round was given the name of ‘Batch 2.’ The chronogram in Figure 1 illustrates the timing of the experiment.

### 2.5. Macroscopic Evaluation

During sacrifices, pictures were taken of all operated knees using an iPhone camera from different angles for the evaluation of macroscopically visible damage in the following parts of the joint surfaces: lateral femoral condyle, medial femoral condyle, lateral tibial plateau, and medial tibial plateau. Damage to these sections in central cartilage was ranked from 0 to 4, in increasing order of severity, according to the Osteoarthritis Research Society International (OARSI) histopathology recommendations for goats and sheep [41], where stage 0 is indicative of normal aspect; stage 1 means surface roughening; stage 2 indicates fibrillation and fissures; stage 3 is for small erosions to subchondral bone; and stage 4 reports larger erosions to subchondral bone. Four different experts blindly and independently evaluated all knees. Finally, an average score for every knee was calculated, and the scores of the four parts of the bones were summed for a possible final number of 0–16.

### 2.6. X-Ray Images and Assessment

Under sedation, direct digital radiographs of the knees (dorso-palmar view) were obtained before surgery at the indicated times in the chronogram (Figure 1). Next, X-ray pictures were blindly evaluated by four experts using the Kellgren–Lawrence classification criteria for OA [42], where grades range from 0 to 4. Stage 0: no joint space narrowing (JSN); stage 1: doubtful JSN and possible osteophytic lipping; stage 2: definite osteophytes and possible JSN; stage 3: moderate osteophytes, definite JSN, some sclerosis, and possible bone-end deformity; and grade 4: large osteophytes, marked JSN, severe sclerosis, and definite bone-end deformities.

### 2.7. Histological Studies

Tissue samples from operated knees were processed at the Microscopy and Anatomic Pathology Core Unit of the Institute for Health Sciences of Aragon (Zaragoza, Spain). After collecting, trimming, and fixing samples in a neutral-buffered formaldehyde solution (4%), they were processed in a rapid tissue processor (X-PRESS ×50 processor, Sakura, Chiba, Japan) until paraffin-embedded. Once obtained, paraffin blocks were cut in 2.5 μm sections using a rotation microtome (Leica RM2255, Leica Biosytems, Barcelona, Spain), and paraffin sections were taken on glass slides. These slides were air dried at 37 °C, deparaffinized in xylene for 10 min, and rehydrated in a grades series of ethanol and distilled water for 5 min.

For hematoxylin–eosin staining, the nuclei of the cells were stained by immersing in Carazzi’s Hematoxylin solution (Panreac, Barcelona, Spain) for 5 min and then washed. Cell cytoplasm was stained by immersing samples in 1% of eosin yellowish hydroalcoholic solution (Panreac, Barcelona, Spain) for 15 min and dipped once in ethanol (70%) for 30 s. For safranin-O counterstaining, preparations were stained through immersion in 0.1% safranin-O solution (Panreac, Barcelona, Spain) for 5 min. Once finished all staining, sections were dehydrated through immersion in ascending alcohol solutions for 15 s each. Finally, all sections were cleaned with xylene for a few seconds and mounted with a cover-slipping reagent (Leica CV Mount, Leica Biosytems, Barcelona, Spain) using glass coverslips.

Finally, two different operators, using at least two slides from each sample, blindly evaluated the preparations. They were given a score using several classification criteria. For microscopic scoring of synovial changes, the OARSI criteria were followed [41], where different parameters are measured, all of them using a severity scale from 0 to 3:

- Synovial hyperplasia: stage 0: normal (one cell deep only); stage 1: mild focal (2–4 cells deep and <20% area); stage 2: mild diffuse (2–4 cells, >20%) or moderate focal (≥5 cells, <20%); and stage 3: moderate diffuse (≥5 cells, >20%).
- Inflammation: stage 0: normal (occasional immune cell); stage 1: mild focal infiltration; stage 2: moderate, diffuse infiltration; and stage 3: marked, discreet lymphoid aggregates.
- Fibrosis: stage 0: none; stage 1: light, focal collagen staining (<20%); stage 2: heavy focal (<20%) or slight diffuse staining; and stage 3: heave diffuse collagenous staining.
- Vascularity: stage 0: 0–2 vascular elements per 100× field; stage 1: 3–4 vascular elements per 100× field; stage 2: 5–8 vascular elements per 100× field; and stage 3: ≥8 vascular elements per 100× field.

The aggregate score (0–12) for synovial changes was also calculated.

For microscopic scoring of the femoral condyle and the tibial plateau, two different criteria were followed from Glasson et al. [43] and Pritzker et al. [44] (‘Glasson’ and ‘Pritzker’ from now on). Both of them establish an osteoarthritic damage severity from 0 to 6.

Glasson criteria: grade 0: normal; grade 0.5: loss of safranin-O without structural changes; grade 1: small fibrillations without loss of cartilage; grade 2: vertical clefts down to the layer immediately below the superficial layer and some loss of surface lamina; grade 3: vertical clefts/erosion to the calcified cartilage extending to <25% of the articular surface; grade 4: vertical clefts/erosion to the calcified cartilage extending to 25–50% of the articular surface; grade 5: vertical clefts/erosion to the calcified cartilage extending to 50–75% of the articular surface; and grade 6: vertical clefts/erosion to the calcified cartilage extending to >75% of the articular surface.

Pritzker criteria, accumulative abnormalities graded from 0 to 6: grade 0: normal architecture; grade 1: surface intact with abrasion and cell death in superficial zone; grade 2: surface discontinuity; grade 3: vertical fissures or clefts; grade 4: erosion; grade 5: denudation; and grade 6: bone deformation.

### 2.8. ELISA and Multiplex Assays

Synovial fluid samples from several experimental points of the in vivo experiment were collected (pre-op, pre-T1, pre-T2, pre-T3, pre-T4, pre-T5, and end-point; see Section 2.4 above), and the presence and concentration of several cytokines were quantified. Assessments of the sheep inflammatory cytokines IL-1β, IL-6, IL-10, IL-17A, and TNF-α were performed at the Cell Sorting and Cytometry Unit of the Institute for Health Sciences of Aragon (Zaragoza, Spain), following the protocol from the MILLIPLEX^®^ Map Ovine Cytokine/Chemokine panel 1, 96-Well Plate Assay (Merck, Rahway, NJ, USA), and reading samples in a Labscan 100 Luminex (Luminex Corporation, Austin, TX, USA). Note that for IL-10, we could not assess its concentration for the end-point samples, while for IL-17A, only end-point samples were available to assess its quantification.

### 2.9. Statistical Analysis

Two-tailed, unpaired *t*-tests with Welch’s correction were performed in order to evaluate statistical significances between treatment groups with GraphPad Prism 8.1.2 software (San Diego, CA, USA). For all cases, differences were considered significant when the *p* value scored under 0.05.

## 3. Results

### 3.1. Damage Reduction in Tibial Plateau Following Preventive Treatment with the Secretome and LUV-TRAIL

**Figure 2:**
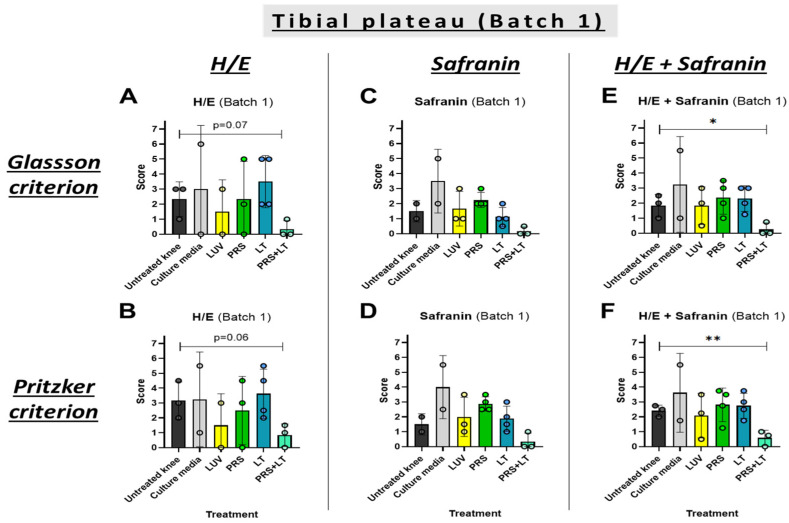
Microscopic evaluation of tibial plateau changes of operated knees from sheep. Graphics show scores from histological preparations with hematoxylin/eosin (**A**,**B**), safranin-O (**C**,**D**), or both (**E**,**F**) stainings of the Batch 1 round of sacrifices. Results were evaluated following either Glasson (**A**,**C**,**E**) [43] or Pritzker (**B**,**D**,**F**) [44] criteria. Each result displayed in H/E + Safranin graphics (**E**,**F**) represents the mean result of H/E and Safranin for every knee and its respective batch. Bars reflect the means ± SD of n = 2–4. *, *p* < 0,05; **, *p* < 0.02. LUV, large unilamellar vesicles; PRS, secretome; LT, LUV-TRAIL.

Beginning with the histological studies, knees were collected from sheep in two different rounds. The first one, named ‘Batch 1,’ corresponds to the first half of the sheep, after meniscectomies and 6 months of observation for OA symptoms, in which sheep have been treated with five rounds of treatments (every two weeks) in one knee, leaving the other without treatment. The second one, named ‘Batch 2,’ corresponds to the remaining sheep, which received one last treatment and were left untreated for three months and then sacrificed (see Section 2.4). We explored the effect of either the secretome, LUV-TRAIL, or both combined in preventing or mitigating damage in the edges of the bones that link the knee joint (the tibial plateau and the femoral condyle). Knee samples were processed and stained with H/E and safranin-O and evaluated. Two different criteria, Glasson [43] and Pritzker [44], were used to increase the robustness and reproducibility of our data (see Section 2.7). Results from tibial plateau injury in the first batch of sacrifices are displayed in Figure 2. A clear tendency towards a healthier cartilage was obtained when knees were treated with the combined therapy after meniscectomies. In fact, when combining information from both types of staining, differences were statistically significant compared to untreated knees (Figure 2E,F). Remarkably, the mean score of all samples from this group was below one, indicating preservation of optimal cartilage morphology. In contrast, single treatments or control groups did not produce any other significant changes.

The remaining sheep were given one last round of treatments and, three months later, sacrificed, and the evolution of the knees was studied (Batch 2). For this group, tibial plateau damage was evaluated separately in the medial and lateral compartments scores using safranin-O counterstaining only.

**Figure 3:**
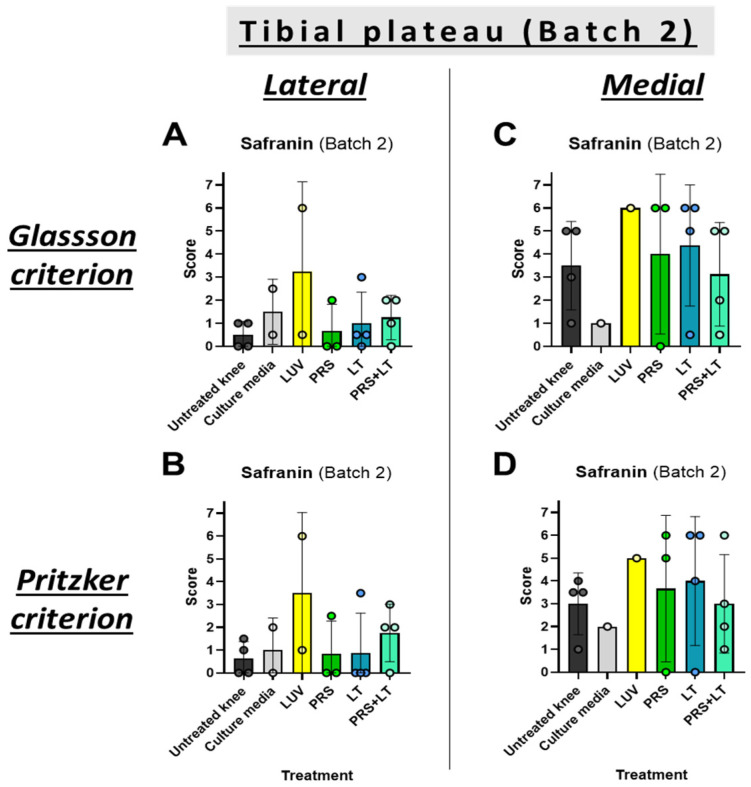
Microscopic evaluation of lateral (**A**,**B**) and medial (**C**,**D**) tibial plateau changes of operated knees from sheep. Graphics show scores from histological preparations with safranin-O stainings of the Batch 2 round of sacrifices. Results were evaluated following either Glasson (**A**,**C**) or Pritzker (**B**,**D**) criteria. Bars reflect the means ± SD of n = 1–4. LUV, large unilamellar vesicles; PRS, secretome; LT, LUV-TRAIL.

Contrary to what was obtained in Batch 1, the combined treatment did not stop damage to any of the sub-sections of the tibia. Equally, and similar to the previous results, secretome or LUV-TRAIL groups did not improve the outcome of this bone, either (Figure 3). Thus, the combination of these two treatments effectively prevented cartilage erosion during the initial steps of OA development (Batch 1). However, when treatment stopped, and due to the severity of the meniscectomy model, OA signs finally arose (Batch 2).

**Figure 4:**
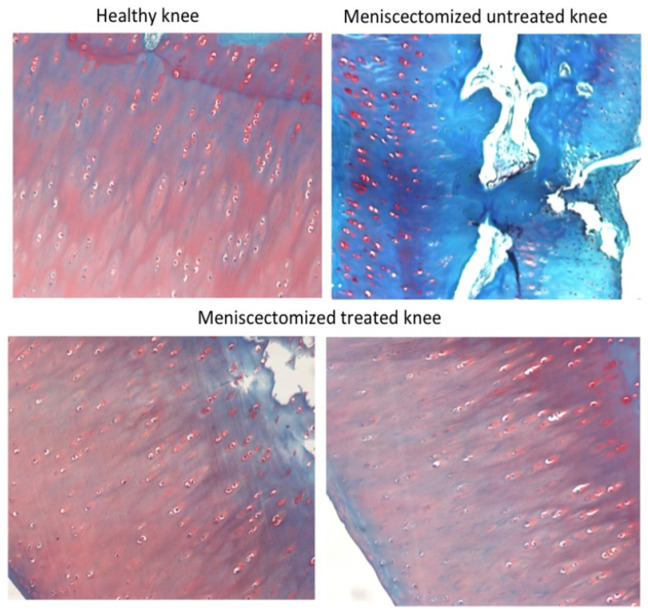
Representative histological safranin-O counter-staining images of tibial plateaus from a healthy sheep knee, from a meniscectomized untreated right knee, and from a meniscectomized left knee treated with a combination of the secretome with LUV-TRAIL, as indicated. The meniscectomized untreated and treated knees correspond to the same animal, included in Batch 1. Magnification was 100-fold.

In Figure 4 are shown representative histological tibial plateau images from a healthy knee or from knees from the same meniscectomized animal (belonging to Batch 1), untreated (right knee), or treated with the combination of the secretome with LUV-TRAIL (left knee). As it can be observed, the sample from the healthy control knee exhibited normal hyaline cartilage, and samples from the meniscectomized and treated left knee showed similar architecture to the control knee. On the contrary, the meniscectomized and untreated right knee displayed severe fibrillations and erosions. It also presented a loss of safranin-O staining due to the deterioration of the cartilage and the subsequent decrease in the glycosaminoglycan component.

As in tibial plateaus, femoral condyles were processed in parallel and in the same manner for our histology evaluations. This analysis was not very informative, as there was barely any damage to the femoral condyle in this experimental model, at least at the time points analyzed, with score values in untreated knees below 1; thus, the treatments had no significant effect (Figures S1 and S2).

### 3.2. The Combination of the Secretome and LUV-TRAIL Mitigates Synovial Hyperplasia in Cartilage

**Figure 5:**
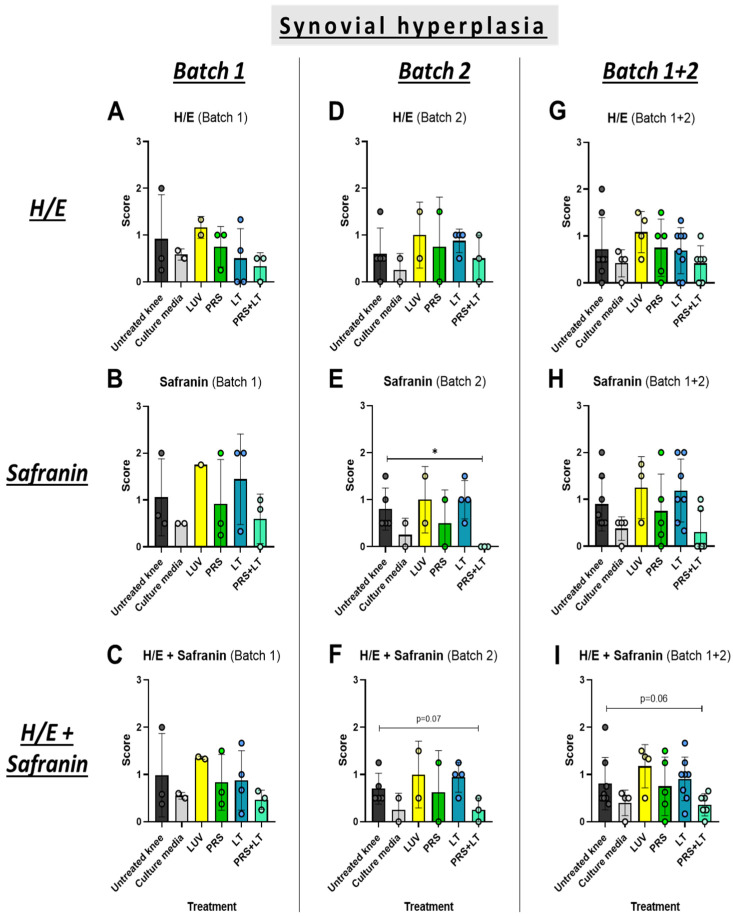
Microscopic evaluation of synovial hyperplasia changes in operated knees from sheep. Graphics show scores from histological preparations with hematoxylin/eosin (**A**,**D**,**G**), safranin-O (**B**,**E**,**H**), or both (**C**,**F**,**I**) stainings for Batch 1 (**A**–**C**), Batch 2 (**D**–**F**), or both (**G**–**I**) rounds of sacrifices. Each result displayed in H/E + safranin graphics (**C**,**F**,**I**) represents the mean result of H/E and safranin for every knee and its respective batch. Bars reflect the means ± SD of n = 1–8. * *p* < 0.05. LUV, large unilamellar vesicles; PRS, secretome; LT, LUV-TRAIL.

Next, we evaluated cartilage or synovial damage, analyzing several parameters: synovial hyperplasia, inflammation, fibrosis, and vascularity. We detected a significant decrease in synovial hyperplasia using the combined therapy in the second batch of sheep when tissues were stained with safranin-O (Figure 5E). In addition, although the differences did not reach significance by a short gap, this positive tendency for the double treatment was also present when the data of H/E and safranin-O were analyzed together for Batch 2 (Figure 5F) or for Batch 1 + Batch 2 (Figure 5I). In contrast, the secretome or LUV-TRAIL alone were not sufficient to improve synovial hyperplasia compared to untreated knees. Therefore, a positive effect of the combination of both treatments on this parameter was induced, which was preserved in Batch 2 after therapy withdrawal.

**Figure 6:**
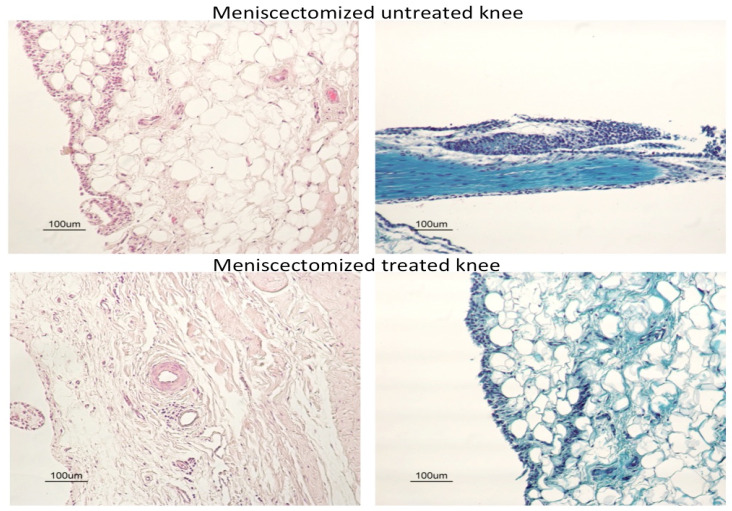
Representative histological images of synovial tissue from meniscectomized untreated or treated knees, as indicated. The treatment was the combination of the secretome with LUV-TRAIL. The untreated and treated knees correspond to animals included in Batch 2. Images at the left correspond to hematoxylin/eosin staining, and images in the right panels correspond to safranin-O counterstainings.

In Figure 6, representative histological synovial tissue images of knees from meniscectomized animals (belonging to Batch 2), untreated, or treated with the combination of the secretome with LUV-TRAIL are displayed. As shown in this figure, synovial hyperplasia was more pronounced in untreated knees when compared to treated ones.

Next, we assessed inflammation signs in our cartilage histologic preparations. Here, we did see one positive tendency towards less inflammation for the combined therapy in the second batch of knees counterstained with safranin-O. Beyond this, none of the other treatment options, time points, or stainings returned any reduction in this parameter (Figure S3). Regarding fibrosis, our treatments, either alone or combined, did not manage to reduce its score compared to the other untreated knees at any of the time points in this experiment (Figure S4). Finally, concerning vascularity, we found a similar situation as in the fibrosis analysis, where scores reflected almost no changes in this parameter following treatments for Batch 1 or 2 sacrifices for any of the staining (Figure S5).

### 3.3. Determination of Inflammatory Cytokines Through Multiplexed ELISA

**Figure 7:**
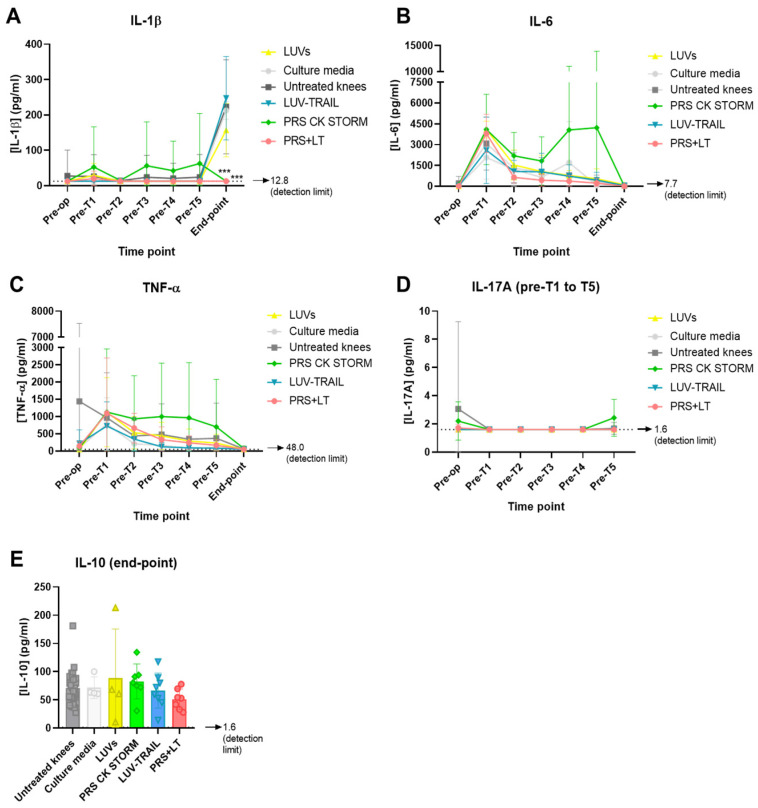
Determination of cytokines through multiplexed ELISA from synovial fluid at several time points: pre-op, pre-T1 to T5, and end-point for IL-1β (**A**), IL-6 (**B**) and TNF-α (**C**); pre-op and pre-T1 to T5 for IL-17A (**D**); and end-point for IL-10 (**E**). Bars and dots represent the means ± SD. ***, *p* < 0.01. LUV, large unilamellar vesicles; PRS, secretome; LT, LUV-TRAIL.

Apart from assessing tissue preparations, we also collected synovial fluid before meniscectomies (‘pre-op’ sample), the first five rounds of treatments (tagged as ‘pre-T1-5’), and prior to sacrifices (named ‘end-point’) (please check Material and Methods and Figure 1 for detailed information) from all operated knees (treated and untreated ones). Then, the presence and quantity of several cytokines were analyzed. In particular, several inflammatory cytokines were measured through multiplexed ELISA: IL-1β, IL-6, TNF-α, IL-17A, and IL-10. The concentration of IL-1β remained very low from the beginning and during treatments for all experimental groups, being close or under detection limit. However, at the moment of sacrifice, most groups suffered a clear increase of this cytokine in the joint (Figure 7A). Given that end-point samples were collected months after the last treatment, it is reasonable to think that a deterioration of the knees during that period developed. Remarkably, the secretome alone or in combination with LUV-TRAIL did not follow this tendency, and IL-1β remained undetectable (Figure 7A), showing a significant positive effect that correlates with the beneficial effect of the combination on synovial hyperplasia.

### 3.4. Macroscopic Observations Showed a Tendency Towards Healthier Joints in LUV-TRAIL-Treated Knees

**Figure 8:**
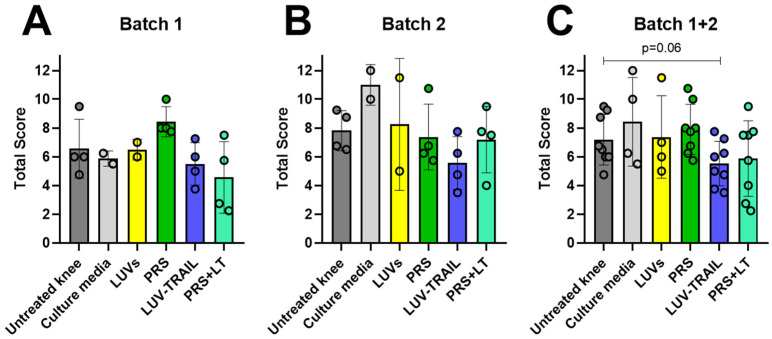
Macroscopic evaluation of operated knee joint’s changes from sheep. Graphics show total scores from pictures of open knee joints, immediately after their sacrifice, for Batch 1 (**A**), Batch 2 (**B**), or both (**C**). Results were evaluated following OARSI recommendations for sheep. Bars reflect the means ± SD of n = 2–8.

**Figure 9:**
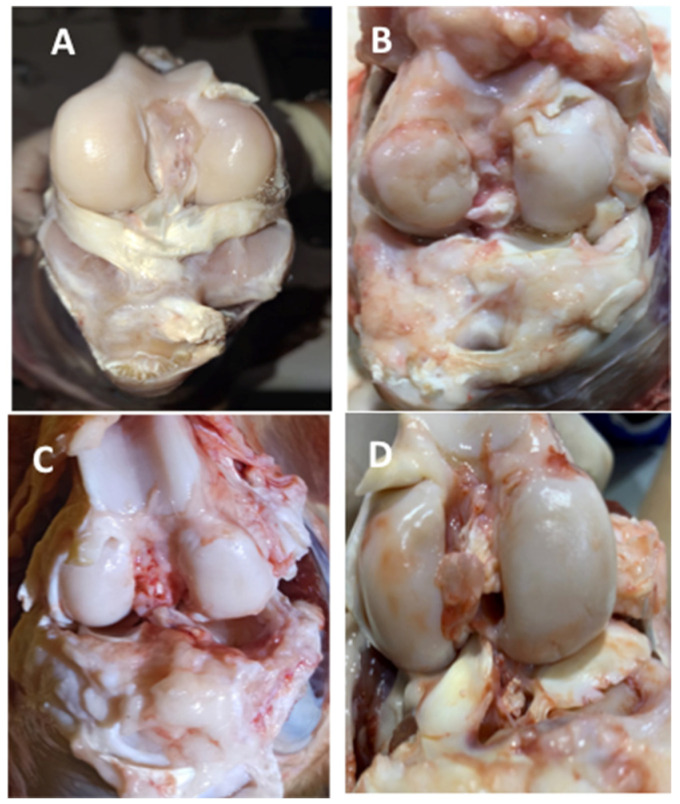
Macroscopic images of sheep knees. (**A**) Control knee from a healthy sheep; (**B**) untreated meniscectomized knee from a sheep in Batch 1; (**C**,**D**) knees from sheep in Batch 1 treated with the combination of the secretome with LUVTRAIL.

Immediately after sheep sacrifices, pictures were taken of all treated knees and several untreated knees, chosen at random. Afterwards, signs of joint damage were evaluated by several experts, following the OARSI criteria [41,43]. Scores were obtained and data were plotted, either by batches or combining results from both, shown in Figure 8. In general, we found a mean reduction in this overall score in LUV-TRAIL groups, alone or in combination with the secretome, especially when the data from both batches were combined (Figure 8). In Figure 9, representative macroscopic images of knees from sheep in Batch 1 are presented. Figure 9A displays a standard knee from a healthy sheep without intervention, with intact menisci and anterior cruciate ligament, while Figure 9B corresponds to an untreated meniscectomized knee, exhibiting a high grade of osteoarthritis (13/16) according to the OARSI histopathology scale. In contrast, Figure 9C,D depict meniscectomized knees treated with the secretome + LUVTRAIL, showing a partial grade of osteoarthritic involvement ranging from three to seven on the OARSI scale.

### 3.5. Post-Mortem Radiographies Suggest Better Outcomes After LUV-TRAIL Treatment

**Figure 10:**
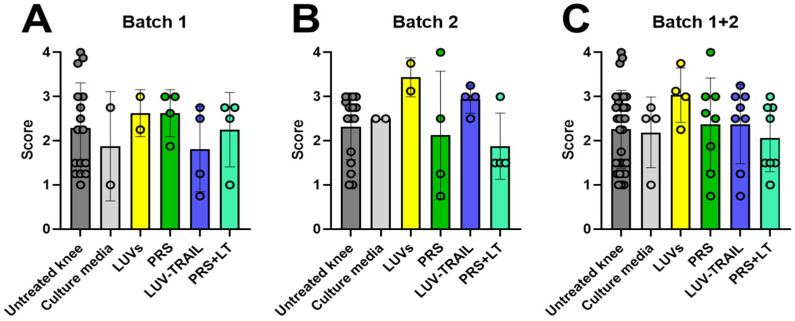
X-ray evaluation of operated knee joint’s changes from sheep. Graphics show total scores from pictures of open knee joints, immediately after their sacrifice, for Batch 1 (**A**), Batch 2 (**B**), or both (**C**). Results were evaluated following Kellgren–Lawrence classification criteria for OA [42]. Bars reflect the means ± SD of n = 2–8. LUV, large unilamellar vesicles; PRS, secretome; LT, LUV-TRAIL.

**Figure 11:**
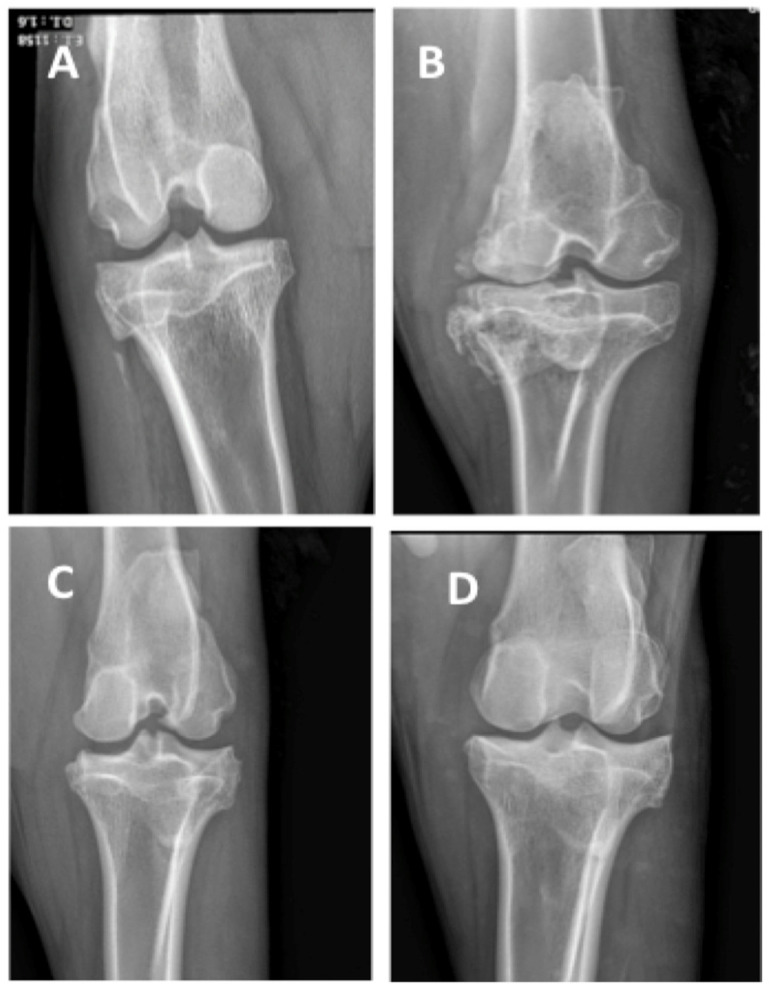
X-ray evaluation of OA in sheep knees. (**A**) Pre-surgical image of the knee from a sheep in Batch 1; (**B**) premortem knee of a meniscectomized untreated sheep from the same batch; (**C**,**D**) premortem radiographs of meniscectomized sheep from Batch 1 after the administration of five treatments with LUV-TRAIL.

Finally, we also performed radiographies for every operated knee at the end-points and evaluated joint damage following Kellgren–Lawrence classification criteria for OA [42]. Similarly to macroscopic observations, X-ray data are presented by batches alone or combined in Figure 10. On one hand, for LUV-TRAIL treatment, graphics suggested that sheep injected with this drug could mitigate their joint space narrowing by the beginning of OA (Figure 10A), but it would eventually happen if not treated repeatedly (Figure 10B), in agreement with data obtained from tibial plateau affectation. In Figure 11, representative macroscopic images of knees from sheep in Batch 1 are shown. Figure 11A displays a knee from a sheep before the intervention, with intact joint space, while Figure 11B corresponds to an untreated meniscectomized knee, presenting the highest grade of osteoarthritis (grade 4) according to the modified Keelgen–Lawrence scale used in this study. In contrast, Figure 11C,D depicts meniscectomized knees from the same batch treated with LUV-TRAIL, showing the lowest treatment radiographic score (1.5).

## 4. Discussion

Aging is considered one of the most common risk factors to develop OA [4]. Despite being true that OA represents a disease that not always appears when aging, progress and technological advances are increasing the population’s lifespan, which inevitably promotes the emergence and prevalence of this disease [2]. In addition, injuries and traumas to joints recurrently happen in people during exercise or by accident, which greatly increase the probability to eventually suffer from this pathology [15]. Thus, OA treatment stands as a field in constant need of finding better therapies to prevent, mitigate, or even eradicate its signs and symptoms. It is not intended, far from it, to overlook the benefits that corticosteroid treatment or joint replacement have provided throughout the decades. They continue to improve the quality of life of OA patients. Nevertheless, despite these efforts, this disease remains incurable, and alternatives need to be found.

In this regard, research worldwide has focused on alleviating the main symptoms of this medical condition: pain, mobility, inflammation, and joint destruction [45]. Similarly to corticosteroids, hyaluronic acid injections have been extensively studied to mitigate pain in knee joints; however, their efficacy and safety profile are currently in question following recent studies [46]. As alternatives, there exist multiple cell-based therapies under investigation. For instance, PRP has been demonstrated to achieve better evaluation scores in short to mid-term therapy [18]; MSCs, possibly through their intrinsic regenerative capacity [3] and/or the release of exosomes [47], have been demonstrated to mitigate OA symptoms [3,48]; and other mixes of cells, such as bone marrow aspirate concentrate or adipose-derived stromal vascular fraction cells, have reported favorable outcomes in the clinic [49,50]. The mechanism of action for these candidates remains still unclear, but polarizing the synovial moiety using products derived from cells or that mimic cell interactions are opening the gate for present and future therapies in OA.

Our proposals are also inspired by this idea of modulating the joint microenvironment through products derived from cells. The secretome described in this work is an innovative treatment that contains plenty of anti-inflammatory signals derived from cell-to-cell paracrine action [36], and LUV-TRAIL imitates physiological responses from immune cells, with prior promising results against rheumatoid arthritis [34]. Given that current therapies are logically oriented and tested on patients already suffering from early-to-late OA, and knowing the usual risk factors for developing this disease, we sought to address the problem before its emergence. Therefore, we began treating our sheep with preventive doses of our treatments after meniscectomies and evaluated their effect in the short and mid-term.

Assessment of tibial plateau damage at our two end-time points exhibited a clear and significant reduction in the first batch of treatments using the secretome in combination with LUV-TRAIL. This means that our combined therapy allowed for avoiding most damage to the tibia during early development of OA in sheep. Evaluations of macroscopic images and radiographies, albeit not reaching statistical significance, also point towards a protective action of this combined treatment in the joint. This beneficial effect was, however, not maintained in the second batch, which did not receive additional treatment in the last 3 months before sacrifice. These data indicate that our treatment can prevent bone erosion and OA development at the early stage, but, due to the severity of the meniscectomy model, once the treatment is withdrawn, cartilage erosion appears. In counterpart, in the other bone member of the joint, the femur, we could not see significant differences in treated vs. untreated knees, probably due to the fact that untreated knees, based on their histological scores, were not as affected as the ones from the tibia. In the literature, not many studies address the evolution of tibial plateau and femoral condyle degeneration during the course of OA. However, interestingly, in a paper by Beuf et al., where the trabecular structure of those two bones was studied to compare several severity stages of OA, faster loss of trabecular spacing in the tibia than in the femur was observed [51]. In addition, other findings suggested that femur cartilage is more resistant to early stages of OA in comparison with tibia cartilage [52]. Even for the meniscectomy sheep model in particular, it has been reported that early OA signs following meniscus trauma initially appear in the cartilage and subchondral bone [53,54]. Biomechanically, it is logical that the tibial plateau suffers from greater erosion, as during knee flexion higher compressive loads are transmitted to this bone compared to the femur [55]. Thus, the fact that the secretome in combination with LUV-TRAIL protected the tibial plateau from early signs of OA is especially relevant and could be recommended to patients with traumatic knee lesions as a preventive therapy.

Results from the histological studies reflected that the combined treatment was actually able to almost nullify the damage in the synovial capsule in terms of synovial hyperplasia as a preventive treatment in agreement with the cartilage erosion results. Notably, this low score was maintained up to 9 months after the first treatment and 3 months after the last one. We also observed a certain tendency in reducing inflammation with the combined therapy, but no effects were observed on fibrosis or vascularity.

In an attempt to clarify results regarding inflammation, several cytokines were measured from synovial fluid at different time points. IL-1β and TNF-α are considered to be the most important pro-inflammatory cytokines involved in OA’s pathogenesis [56]. Regarding IL-1β, a peak of this cytokine emerged at the end-point. This can be explained because stopping treatments for several months may lead to an increase in this pro-inflammatory cytokine. Remarkably, the secretome alone or its combination with LUV-TRAIL completely inhibited the secretion of this cytokine. This observation could explain the tendency towards a lower inflammation profile in histological studies for the combined therapy. In the case of the secretome treatment alone, however, no decrease in inflammation was found.

We are also aware that we are using a ‘cocktail’ of human anti-inflammatory cytokines and factors in a different species, which could limit or vary the response in the animal [57] in comparison with a hypothetical human response. Nevertheless, the secretome described here has already proven to be effective in combating severe systemic inflammation in mice [37], which encouraged us to test its efficacy in OA. Another alternative would be to manufacture this secretome by exclusively co-cultivating ovine anti-inflammatory macrophages with ovine chondrocytes, a procedure that will require previous isolation of both types of cells from the animals.

Soluble TRAIL exhibits limited stability and bioactivity in vivo, which has prompted its delivery via carrier systems or its use in combination therapies. For example, especially in the context of cancer treatment, TRAIL has benefited from combinations with doxorubicin [58,59], flavopiridol [25], bortezomib [60], or, more recently, CDK9 inhibitors [61]. In fact, TRAIL has already proven to effectively inhibit the proliferation of T cells present in rheumatoid synovial fluid [33], and LUV-TRAIL has been shown to reduce synovial hyperplasia and inflammation in a rabbit rheumatoid arthritis model [34]. It is currently well described that during OA’s development, several types of immune cells, including T cells, are attracted to the injured joint and promote inflammation and more damage [14]. Several studies have detected abnormal concentrations of T helper, cytotoxic T lymphocytes, or both at different stages of OA’s development [62]. Yet, the specific pathological mechanism of T cells in OA is still unknown. In this work, apart from its effects in combination with the secretome, LUV-TRAIL alone also displayed some reduction in macroscopic knee damage. The profound effect on synovial hyperplasia observed in the rheumatoid arthritis model could be related to the observation in the present study of the most durable effect of our treatment combination in OA. This could be due to an effect on infiltrating T cells, inhibiting the secretion of cytokines that could induce the proliferation of synoviocytes, or to a direct effect on these last cell types, being a subject of further study. In fact, another study reported that TRAIL effectively reduced inflammation in rheumatoid arthritis through cell-death-independent inhibition of T lymphocytes [63]. It is known that apart from inducing cell death in selected targets, TRAIL is also capable of activating proliferative and differentiation pathways in other cells, such as vascular endothelial cells and skeletal muscle cells, presumably via Akt phosphorylation [28,64].

## 5. Conclusions

The chronicity and degenerative nature of OA are constantly pushing researchers to seek the most suitable treatment to combat this disease. Joint replacement aside, since the discovery of the large impact of inflammation and immune cells in OA’s pathogenesis, most successful therapies are based on controlling or eliminating their effect in the injured joint. Our combined therapy of is no exception. Our experiments have demonstrated that these two treatments combined are capable of improving knee injury in the first stages of OA in our ovine model for several parameters, such as synovial hyperplasia and tibial plateau damage, which are two of the most frequently damaged areas. Other markers, such as synovial inflammation and X-ray and macroscopic images, also presented a tendency to improve. Further studies will help to elucidate how many positive effects this therapy can bring and which is the mechanism of action of this combinatory effect.

## Figures and Tables

**Table 1:** Secretome characterization. Results are shown as the mean of triplicates for every protein in pg/mL.

**MIP-1α**	**SDF-α**	**IL-27**	**LIF**	**IL-1β**	**IL-2**	**IL-4**	**IL-5**
81.32	247.56	<21.41	20.78	<2.16	<7.21	<10.49	<9.90
**IP-10**	**IL-6**	**IL-7**	**IL-8**	**IL-10**	**PIGF-1**	**Eotaxin**	**IL-12 p70**
13.87	403.78	<0.99	268.45	1.99	<1.71	2.56	<4.71
**IL-13**	**IL-17A**	**IL-31**	**IL-1RA**	**SCF**	**RANTES**	**IFN-γ**	**GM-CSF**
<3.58	<2.27	<9.21	63,389.96	<3.58	<2.27	<9.21	<2.72
**TNF-α**	**HGF**	**MIP-1β**	**IFN-α**	**MCP-1**	**IL-9**	**VEGF-D**	**TNF-β**
12.89	371.56	132.87	<0.45	2876.34	<2.89	<0.79	<5.69
**NGF-β**	**IGF-1**	**BDNF**	**GRO-α**	**IL-1α**	**IL-23**	**IL-15**	**IL-18**
<6.14	<1.78	<0.34	10.21	<0.61	<6.14	<1.78	<0.34
**IL-21**	**FGF-2**	**IL-22**	**PDGF-BB**	**VEGF-A**	**TIMP-1**	**MMP-3**	**MMP-1**
<6.37	<2.72	<18.07	14.87	<6.37	63,389.96	<18.07	14.87

## Data Availability

The datasets generated during and/or analyzed during the current study are available from the corresponding authors upon reasonable request.

## Supplementary Materials

The following supporting information can be downloaded at https://www.mdpi.com/article/10.3390/pharmaceutics18020193/s1, Figure S1: Microscopic evaluation of femoral condyle changes of operated knees from sheep; Figure S2: Microscopic evaluation of lateral and medial femoral condyle changes of operated knees from sheep; Figure S3: Microscopic evaluation of synovial inflammation changes of operated knees from sheep; Figure S4: Microscopic evaluation of synovial fibrosis changes of operated knees from sheep; Figure S5: Microscopic evaluation of synovial vascularity changes of operated knees from sheep.
