# Supplementary material for: The Combination of Immunomodulatory Secretome and Liposome-Bound TRAIL Improves Knee Osteoarthritis Symptoms in an Ovine Model
Source: Pharmaceutics. 2026 Feb 2;18(2):193. doi: 10.3390/pharmaceutics18020193 (PMC12944125; doi:10.3390/pharmaceutics18020193)
Supplement: Supplementary file 1 [file pharmaceutics-18-00193-s001.zip › pharmaceutics-4078137-supplementary.pdf]

Supplemental Figures

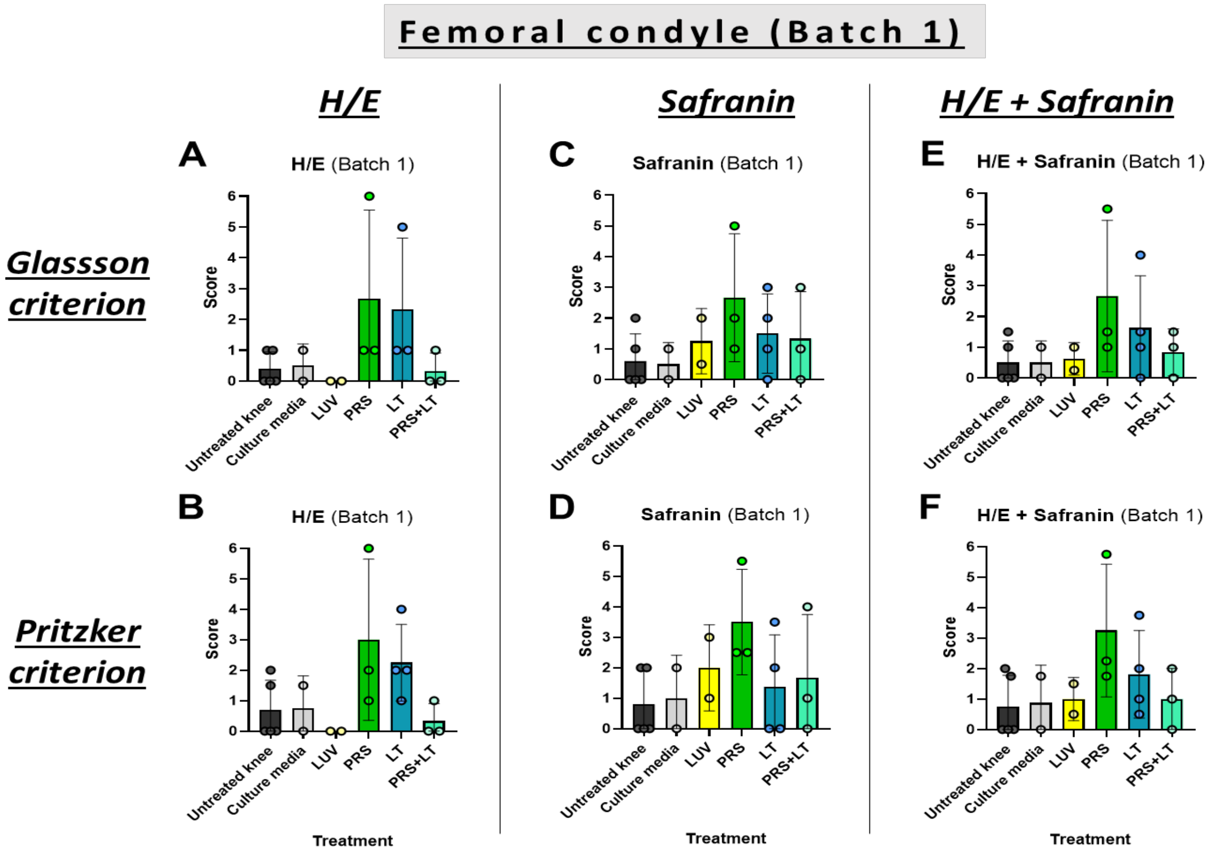

Figure S1. Microscopic evaluation of femoral condyle changes of operateds knees from sheep. Graphics show scores from histological preparations with hematoxylin/eosin (A, B), safranin-O (C, D) or both (E, F) stainings of Batch 1 round of sacrifices. Results have been evaluated following either Glasson (A, C, E) or Pritzker (B, D, F) criteria. Each result displayed in H/E + Safranin graphics (E, F) represents the mean result of H/E and Safranin, for every knee and its respective Batch. Bars reflect the mean +/- SD of n=2-4.

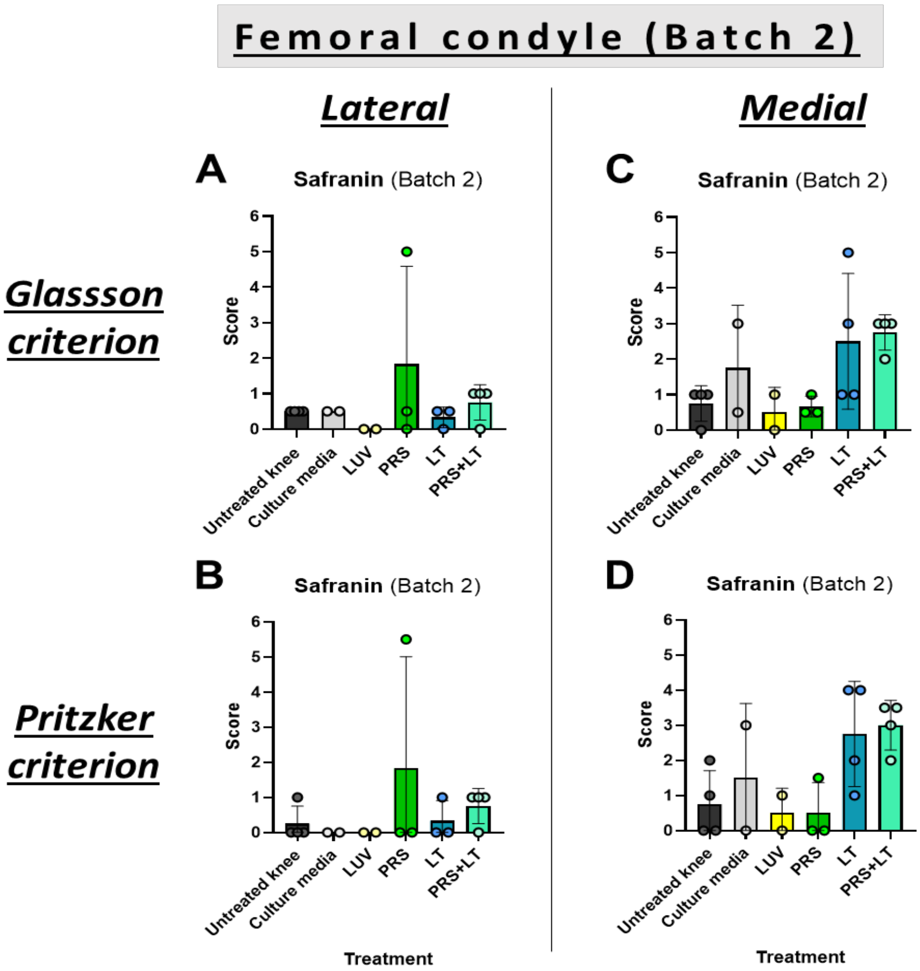

Supplemental Figure S2. Microscopic evaluation of lateral and medial femoral condyle changes of operated knees from sheep. A,B, lateral. C,D, medial. Graphics show scores from histological preparations with safranin-O stainings of Batch 2 round of sacrifices. Results have been evaluated following either Glasson (A, C) or Pritzker (B, D) criteria. Bars reflect the mean +/- SD of n=1-4.

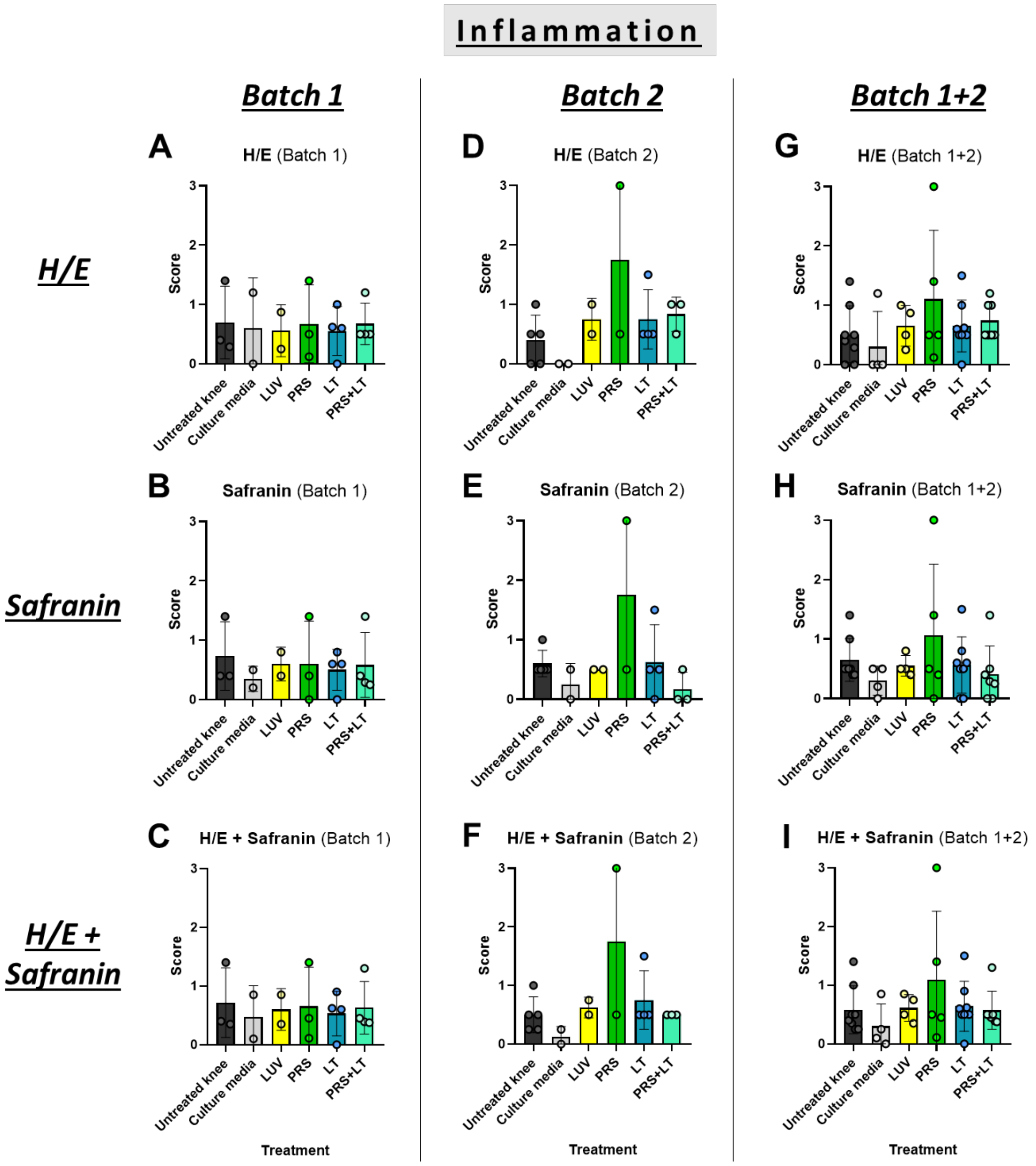

Supplemental Figure S3. Microscopic evaluation of synovial inflammation changes of operated knees from sheep. Graphics show scores from histological preparations with hematoxylin/eosin (A, D, G), safranin-O (B, E, H) or both (C, F, I) stainings of Batch 1 (A, B, C), Batch 2 (D, E, F) or both (G, H, I) rounds of sacrifices. Each result displayed in H/E + Safranin graphics (C, F, I) represents the mean result of H/E and Safranin, for every knee and its respective Batch. Bars reflect the mean +/- SD of n=2-8.

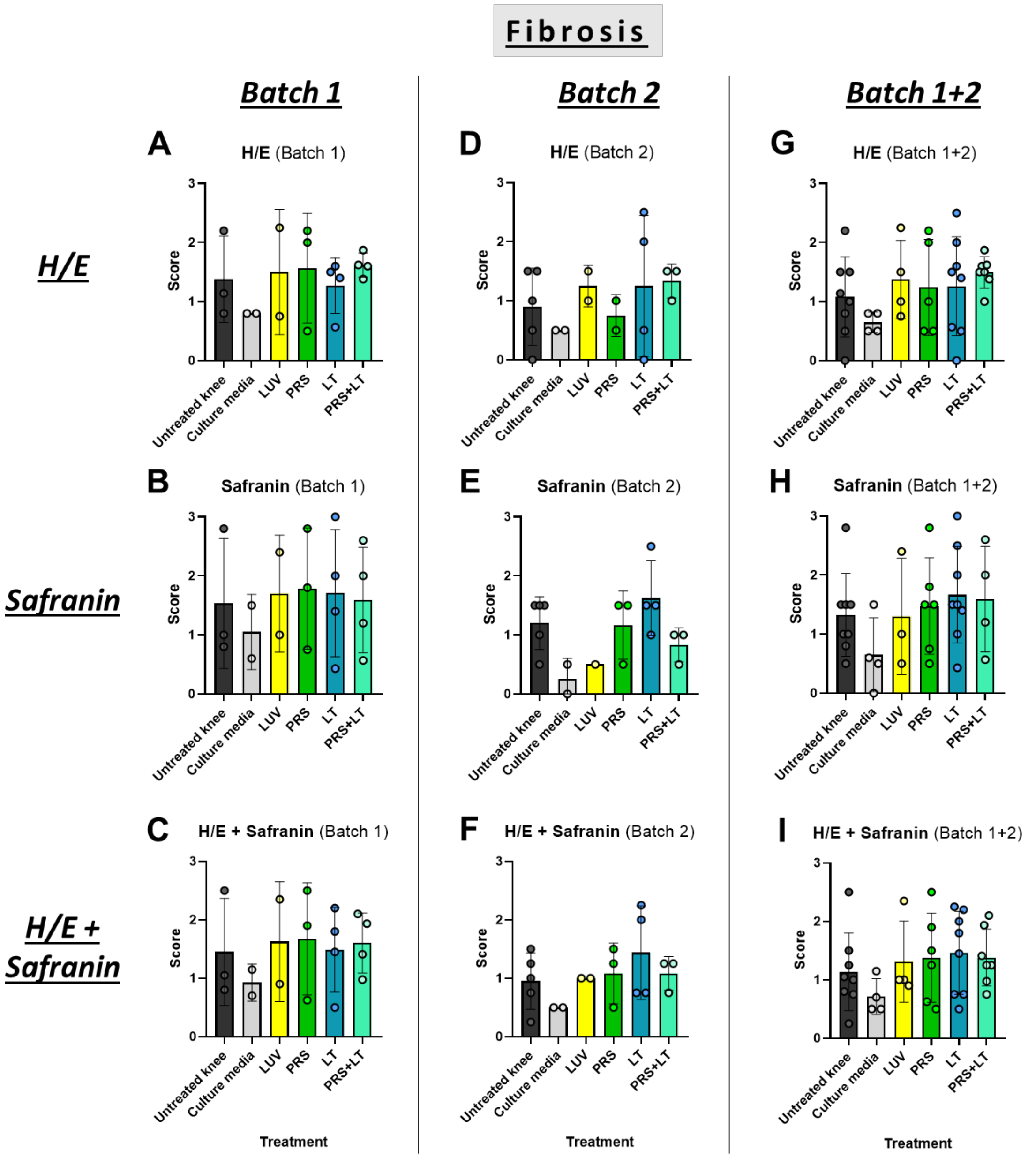

Supplemental Figure S 4. Microscopic evaluation of synovial fibrosis changes of operated knees from sheep. Graphics show scores from histological preparations with hematoxylin/eosin (A, D, G), safranin-O (B, E, H) or both (C, F, I) stainings of Batch 1 (A, B, C), Batch 2 (D, E, F) or both (G, H, I) rounds of sacrifices. Each result displayed in H/E + Safranin graphics (C, F, I) represents the mean result of H/E and Safranin, for every knee and its respective Batch. Bars reflect the mean +/- SD of n=1-8.

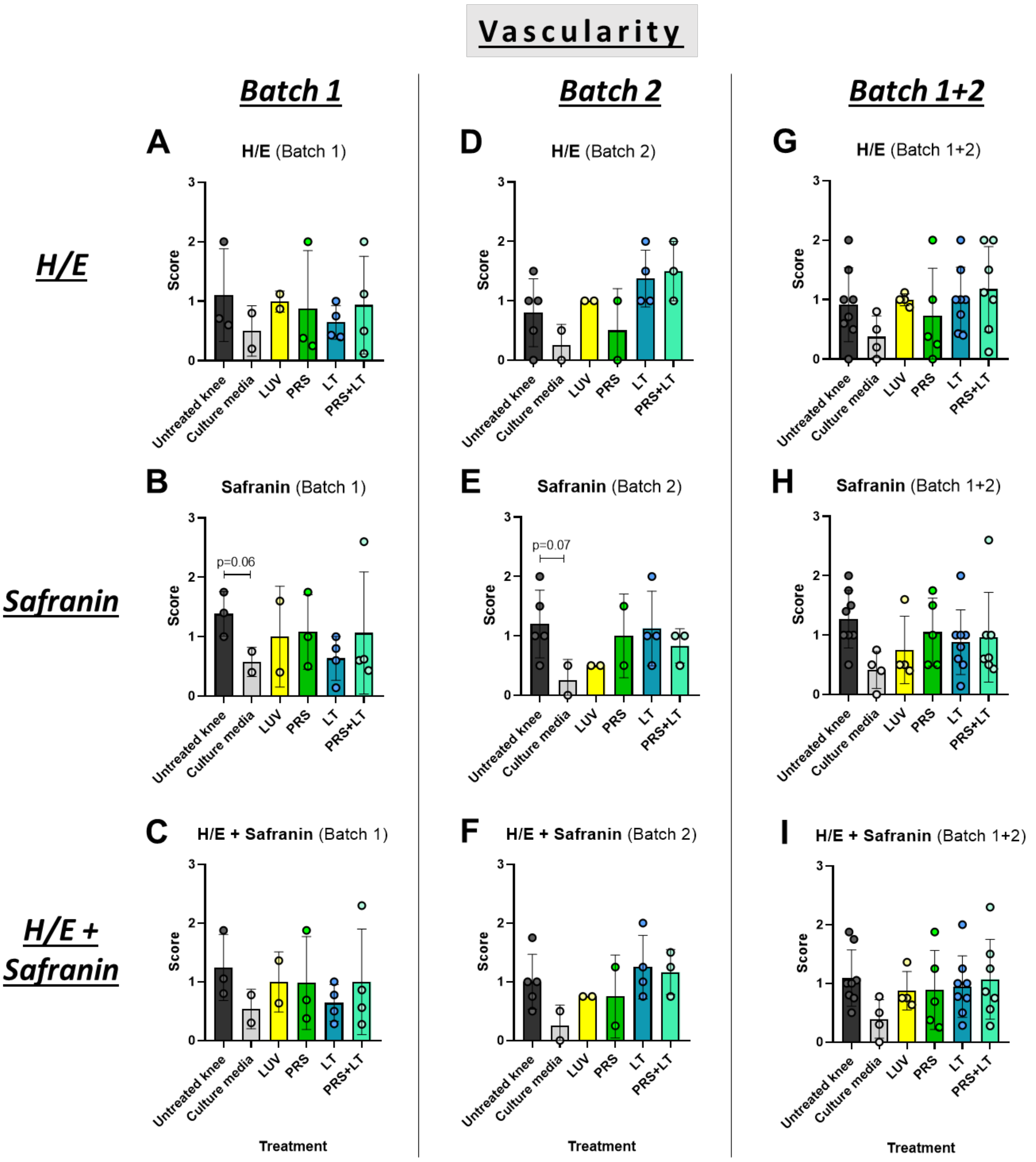

Supplemental Figure S 5. Microscopic evaluation of synovial vascularity changes of operated knees from sheep. Graphics show scores from histological preparations with hematoxylin/eosin (A, D, G), safranin-O (B, E, H) or both (C, F, I) stainings of Batch 1 (A, B, C), Batch 2 (D, E, F) or both (G, H, I) rounds of sacrifices. Each result displayed in H/E + Safranin graphics (C, F, I) represents the mean result of H/E and Safranin, for every knee and its respective Batch. Bars reflect the mean +/- SD of n=2-8.
